# Immersive virtual reality fitness games for enhancement of recovery after colorectal surgery: study protocol for a randomised pilot trial

**DOI:** 10.1186/s40814-022-01213-x

**Published:** 2022-12-13

**Authors:** Sebastian Wolf, Johannes Zanker, Florian Sommer, Dmytro Vlasenko, David R. M. Pinto, Michael Hoffmann, Matthias Anthuber, Matthias C. Schrempf

**Affiliations:** Department of General, Visceral and Transplantation Surgery, University Hospital Augsburg, Stenglinstrasse 2, 86156 Augsburg, Germany

**Keywords:** Colorectal cancer, Virtual reality, Rehabilitation, Pilot trial, Fitness games, Surgical oncology

## Abstract

**Background:**

Physical inactivity after surgery is an important risk factor for postoperative complications. Compared to conventional physiotherapy, activity-promoting video games are often more motivating and engaging for patients with physical impairments. This effect could be enhanced by immersive virtual reality (VR) applications that visually, aurally and haptically simulate a virtual environment and provide a more interactive experience. The use of VR-based fitness games in the early postoperative phase could contribute to improved mobilisation and have beneficial psychological effects. Currently, there is no data on the use of VR-based fitness games in the early postoperative period after colorectal surgery.

**Methods:**

This pilot trial features a single-centre, randomised, two-arm study design with a 1:1 allocation. Patients undergoing elective abdominal surgery for colorectal cancer or liver metastases of colorectal cancer will be recruited. Participants will be randomly assigned to an intervention group or a control group. Patients randomised to the intervention group will perform immersive virtual reality-based fitness exercises during their postoperative hospital stay. Feasibility and clinical outcomes will be assessed.

**Discussion:**

Early mobilisation after surgery is crucial for reducing many postoperative complications. VR-based interventions are easy to use and often inexpensive, especially compared to interventions that require more medical staff and equipment. VR-based interventions could serve as an alternative or complement to regular physiotherapy and enhance mobilisation after surgery. The proposed pilot study will be the first step to evaluate the feasibility of VR-based interventions in the perioperative period, with the aim of improving the postoperative rehabilitation of cancer patients.

**Trial registration:**

The trial has been registered in the German Clinical Trials Register (DRKS) Nr. DRKS00024888, on April 13, 2021, WHO Universal Trial Number (UTN) U1111-1261–5968.

**Supplementary Information:**

The online version contains supplementary material available at 10.1186/s40814-022-01213-x.

## Background

### Early postoperative rehabilitation

Restoring a patient’s physical fitness in the early postoperative period is of particular importance to reduce the risk of poor functional outcomes and complications such as respiratory tract infections and venous thromboembolism [1–3]. In most solid tumour diseases, surgical resection is the most important component of curative cancer therapy. Cancer patients often suffer from fatigue, decreased strength, pain, insomnia and psychosocial stress [4–10]. Surgery can further reduce a patient's fitness and reduce the quality of life.

Prolonged bed rest after the surgery is an important risk factor for postoperative complications and morbidity. Therefore, early mobilisation after surgery is strongly recommended by the Enhanced Recovery after Surgery (ERAS®) Society for most tumour entities [3, 11, 12]. In general, mobilisation of a patient includes activities such as sitting, standing and walking or passive exercises, which are started on the day of surgery and aim to reduce muscular and cardiovascular deterioration and prevent complications due to immobility. Physiotherapy in the early postoperative period plays an important role in achieving this goal.

Lack of staff and mobilisation support have been identified as causes of inadequate physiotherapy and mobilisation in patients undergoing abdominal surgery [13–15]. An Australian study showed that the durations of upright mobilisation were 3.0, 7.6, 13.2 and 34.4 in the first 4 days after upper abdominal surgery [13]. These figures differ greatly from the ERAS® recommendations [3, 12]. Patients undergoing major abdominal surgery require intensive mobilisation support as most of these patients are dependent on assistance in the first few days after surgery, and the need for support is likely to increase in the future as the general surgery population ages [13, 15]. Providing adequate physiotherapy in this patient group is particularly time-consuming and requires a significant amount of staff.

In addition, patient-related factors such as fatigue and non-compliance can result in the amount of exercise performed being significantly less than the amount of physiotherapy provided [8, 10, 16].

Activity-promoting video games are a subset of video games that have gained popularity over the last 15 years. In these games, the player must engage in various forms of physical activity or sports. While playing, energy consumption and heart rate increase in a similar way to physical activity of similar intensity [17, 18].

Although these games were developed for mass entertainment, they have attracted research interest because of their potential applications in the prevention and rehabilitation of a wide variety of diseases [17, 19–23]. In a rehabilitation or exercise setting, fitness games have the advantage over conventional rehabilitation and fitness exercises that they are actual games that evoke fun and enjoyment, which can increase motivation and compliance [24, 25]. Therefore, the use of VR-based fitness games for early postoperative rehabilitation could have additional positive effects on psychological well-being. Compared to conventional physiotherapy, fitness games are in many cases at least as effective and often more motivating and engaging for patients with physical impairments [26].

### Virtual reality

Virtual reality (VR) is an immersive and interactive technology that visually, aurally and haptically simulates a virtual environment and creates a sense of presence in that environment [27–29]. VR is being studied in various clinical settings and has attracted much attention as a low-cost and effective intervention, particularly in psychiatry [30].

The use of VR-based fitness games in the early postoperative phase could contribute to an improved mobilisation of patients and thus reduce the risk of postoperative complications. The improvement in mobilisation could be due to improved compliance and increased motivation, but also because after instruction and successful learning of the game, patients could perform exercises independently, thus reducing the need for staff. This could increase the daily duration of mobilisation. Accelerated postoperative rehabilitation could also have a positive impact on the length of stay. In addition, VR-based fitness games could have a positive effect on the well-being and quality of life of patients, as they can create fun and enjoyment, as well as a brief distraction from current worries, anxieties and the hospital environment.

### Rationale for a pilot trial

Currently, there are no data on the use of VR-based fitness games in the early postoperative period after colorectal surgery. There are no data available to estimate the effect size and thus enable a high-quality sample size calculation. Therefore, this pilot study aims to investigate the clinical and feasibility endpoints of the proposed intervention. Our aim is to assess, whether the design and protocol used will prove effective in terms of patient recruitment, patient compliance and patient acceptance of the intervention, and whether the intervention will have an impact on quality of life, general health, sleep, patient satisfaction, length of stay and complication rates.

## Methods

### Trial design and study population

**Fig. 1:**
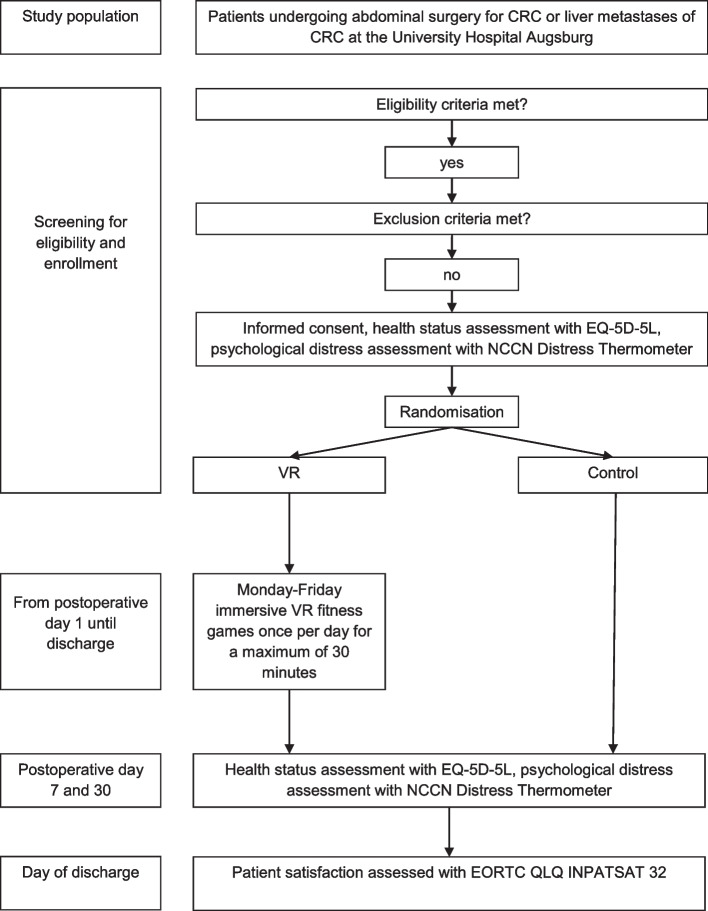
Trial flow chart

This pilot trial features a single-centre, randomised, two-arm study design with a 1:1 allocation. The flowchart of the study design is shown in Fig. 1. Patients undergoing elective abdominal surgery for colorectal cancer or liver metastases of colorectal cancer will be recruited. Participants will be randomized to the intervention group or a control group that receives the standard treatment. Patients randomized to the intervention group will conduct virtual reality-based fitness exercises. This trial is conducted at the department of general-, visceral- and transplantation surgery at the University Hospital Augsburg.

### Informed consent

All patients must provide written informed consent prior to participation. Patients who are unable to give consent, patients under legal guardianship and patients participating in another intervention study that has a potential impact on the endpoint of this study will be excluded from participation.

### Outcomes

#### Feasibility outcomes

The participation rate is the proportion of participants who meet the inclusion criteria and agree to participate in the study. The recruitment rate is the number of participants recruited per month. The retention rate is the number of participants who completed the total number of planned interventions.

- Proportion of interventions carried out in relation to planned interventions for each participant.
- Compliance with the intervention is measured as the percentage VR sessions started compared to the number of sessions offered.
- Rate of discontinued interventions and reasons for discontinuation.
- Compliance with the questionnaire completion is measured as the proportion of questionnaires completed by the set deadlines.
- Number of deviations from the study protocol (e.g. interventions not carried out or carried out differently than described in the study protocol, questionnaires not handed out or not completed) and reason for deviation from the study protocol (patient-related, staff-related, organisational).

#### Clinical outcomes

- Difference in health status assessed with the EQ-5D-5L health questionnaire [31] preoperatively, 7 days after surgery and 30 days after surgery.
- Difference in the number and severity of postoperative complications between the study groups the Clavien-Dindo classification and *Comprehensive Complication Index*® (CCI) [32, 33]
- Difference in length of hospital stay between the two study groups.
- Difference in psychosocial distress preoperatively, at 7 days and at 30 days, assessed with the NCCN Distress Thermometer [34].
- Difference in patient satisfaction at discharge assessed with the EORTC IN-PATSAT 32 [35].

### Interventions

#### Virtual reality (VR) fitness group

On weekdays, each participant in the VR Fitness Group is offered one VR-based activity-promoting fitness session using an immersive virtual reality fitness game until discharge with a maximum duration of 30 min. The sessions will be held in addition to the in-house physiotherapy standard that all patients receive after surgery. Heart rate and blood pressure are measured before each session and 10 min after the session begins to ensure an optimal heart rate for moderate-intensity exercise and to avoid fatigue.

All VR sessions are conducted in a seated position, either with the patient in bed or with the patient on the edge of the bed depending on the patient’s health and preference.

The first training session is conducted entirely under observation by a member of staff or a doctoral student from Augsburg University Hospital. For all subsequent training sessions, the first 10 min are performed under supervision. Participants who are able to continue their sessions on their own or do not require additional support are allowed to continue training on their own after the first 10 min until the end of the session.

The VR headset streams wirelessly to an external device during the first session to monitor the correct execution of the exercises and to guide the participants. This is to ensure that study participants understand the games and perform the exercises correctly. Study participants will also be guided in subsequent sessions as needed.

Only enjoyable content and no depiction of violence or brutal content will be used. Patients perform tasks such as rowing or cycling in a virtual environment as part of the training sessions. The performance of these activities requires the active movement of the upper body, arms, shoulders and hands.

The fitness sessions will be conducted using the Holofit app by Holodia (Holodia, Strasbourg, France) on an Oculus Quest 2 (Facebook Technologies LLC, Menlo Park, U.S.A) device with an Oculus controller in each hand.

##### Exercise intensity

We intend to perform a moderate-intensity exercise. According to the recommendations of the American Heart Association (www.heart.org), moderate exercise is intended to achieve a heart rate range between 50 and 70% of the maximum heart rate.

The target heart rate range is calculated as follows:

- (220—age) × 0.5 to (220—age) × 0.7
- e.g. for a 70-year-old person 75–105 beats per minute
- e.g. for a 50-year-old person 85–119 beats per minute

The training intensity can be increased up to 75% of the maximum heart rate for participants who are fit enough. The prerequisite for this is that 3 consecutive 30-min sessions have been completed successfully and are free of symptoms. The heart rate is measured before the start of the training and 10 min after the start of the training to ensure an appropriate training intensity.

#### Control group

Patients in the control group receive the in-house physiotherapy standard.

#### Physiotherapy

Physiotherapy is provided by physiotherapists. Both groups receive the same amount of physiotherapy, which includes passive and active mobilisation in bed, standing next to the bed, assisted walking and demonstration of breathing exercises. The physiotherapists have no knowledge of a particular patient’s group assignment and study participation. The target daily duration of physiotherapy is 20 to 30 min, and in individual cases, shorter physiotherapy is performed depending on the general condition of the patient. As soon as the patient is able to walk on level ground for 5 min, can climb one flight of stairs without assistance and there are no pulmonary symptoms, physiotherapy and breathing therapy are terminated.

#### Adverse events

The study involves a low-risk intervention. Special considerations were made in advance to increase the safety of the participants by limiting the maximum age of the participants, the intensity of the exercises and the conduct of the exercises in a seated position. All adverse events will be collected and evaluated.

### Inclusion criteria and exclusion criteria

#### Inclusion criteria

Patients undergoing abdominal surgery due to colorectal carcinoma or liver metastases of colorectal carcinoma at Augsburg University Hospital. Patients must be at least 18 to a maximum of 75 years of age, capable of informed consent, not under legal guardianship and must provide written informed consent. If possible, informed consent will be obtained before the operation in the case of elective or urgent operations. In the case of emergency surgery, the patient will be offered trial participation on postoperative day 1 or as soon as the patient is capable of giving informed consent.

#### Exclusion criteria

Patients who are incapable of giving consent, underage patients, pregnant patients and patients participating in another intervention study that could have a possible influence on the endpoints of this study are excluded from participation. Other exclusion criteria include a history of dementia, schizophrenia, hallucinations, panic attacks or epileptic seizures. Wearers of pacemakers or defibrillators as well as patients taking neuroleptics or antiepileptic drugs are excluded from the study. Patients with active alcohol and/or drug abuse will also be excluded from the study. Further exclusion criteria are heart failure NYHA ≥ 3, COPD ≥ Gold 3, myocardial infarction within the last 3 months, untreated cardiac or pulmonary disease and musculoskeletal conditions that limit the age-appropriate range of motion of the upper body, arms, shoulders and neck.

To ensure adequate exercise capacity, patients who are unable to climb 1 floor without interruption preoperatively (MET (Metabolic Equivalent Threshold) ≥ 4) and are unable to perform light housework/gardening are excluded [36]. Recent studies show that exercise-enhancing video games that require light to moderate exertion correspond to a MET of 2.3 to 3.8 [37]. If there is any doubt about the patient’s statement, the ability to climb stairs is verified under supervision.

#### Secondary exclusion from the VR group

For patients who develop postoperative delirium, the intervention will be paused until the patients are consistently symptom-free and fully orientated for 24 h without drug delirium therapy. Patients with a reduced vigilance will not receive the VR fitness intervention as long as vigilance reduction persists. The VR fitness intervention will be paused while a participant is physically unable to sit in bed or at the side of the bed for 30 min. A new diagnosis of any of the conditions listed under the exclusion criteria will result in exclusion from the intervention group. New onset and continued use of any of the medications listed under the exclusion criteria will result in exclusion from the VR fitness group.

### Methods against bias

#### Randomisation

The subjects will be randomised preoperatively to one of the two groups (VR fitness/control) in a concealed fashion. Study randomization will be performed through Study Randomizer (2017), a web-based randomization service, after all inclusion and exclusion criteria have been reviewed [38]. Allocation to the study groups is done in a 1:1 ratio. Block randomisation will be performed to ensure equal-sized groups. The size of the individual blocks will only be disclosed after the study has been completed so as not to allow prediction of group allocation. A sufficient number of subjects will be recruited according to the sample size calculation to prevent random errors and to ensure sufficient power to test the hypothesis of the primary endpoint. Randomisation is done by the staff of Augsburg University Hospital who are not involved in data collection and care of the study patients.

#### Blinding

Due to the study design, the patients and the study staff conducting the VR sessions are not blinded. However, the individuals directly involved in the treatment of patients are blinded to the intervention. The personnel assessing the clinical endpoints that are not collected during the VR sessions are blinded to the intervention.

#### Confounding factors

Performance bias is reduced by using standardised guidance for study staff.

### Sample size calculation

The feasibility study is being carried out with the aim to investigate the clinical and feasibility endpoints of the proposed intervention. Since there is no existing data to enable precise sample size calculations, we adhered to recommendations for sample size calculations in pilot trials. The current postoperative mean length of stay in our own patient population is approx. Thirteen days with a standard deviation of approx. 9.5. A targeted reduction in the length of stay by 2 days would result in a standardized effect size of 0.21. Whitehead et al. recommend a case number of *n* = 28 per group for pilot studies with a standardized effect size of 0.2 and a later planned study with a power of 90% [39]. In order to account for dropouts, e.g. due to non-completed questionnaires or study exclusions, 3 additional patients per group will be recruited. This results in a total number of 62 patients (31 per group).

### Data collection

All data will be documented in hard copy case report forms. The completed CRFs will be reviewed by one of the investigators or an authorized sub-investigator. All data collected according to the study protocol will be manually transferred from the case report forms to an electronic SPSS file (IBM, Armonk NY, USA). Regular reviews of the correct data transfer are conducted by assessors at the study site. The electronic data will be stored in a protected folder on a server at University Hospital Augsburg. Paper-based data are stored in a locked office at the study site.

### Pseudonymisation

Data evaluation is carried out in a pseudonymised form. For this purpose, a randomly generated numerical four-digit code is assigned to each participant. Access to the original data and the pseudonymisation lists is restricted to the staff of the Department of General-, Visceral and Transplant Surgery at the University Hospital Augsburg. The data will be deleted as soon as they are no longer used for research.

### Data analysis plan

Continuous data will be presented as mean ± standard deviation or median with an interquartile range, depending on the distribution. Categorical data will be presented as numbers with percentages. Approximately, normally distributed continuous variables will be compared using the independent *t* test. Non-normally distributed continuous variables will be compared using the Mann–Whitney *U* test. Categorical data will be compared using the *χ*^2^ test. Fisher’s exact test will be used for categorical data if the requirements for *χ*^2^ test are not met. A two-sided *P* < 0.05 is considered significant.

The basic questionnaire used for this study is the validated and standardised EQ-5D-5L health questionnaire of the EuroQol Research Foundation. It will be scored and reported as recommended in the EQ-5D-5L user guide. A questionnaire with additional questions on patient satisfaction (EORTC IN-PATSAT 32) will also be analysed.

## Discussion

Postoperative immobilisation and prolonged bed rest are important risk factors for postoperative surgical and non-surgical complications. As many patients require significant support after abdominal surgery, the provision of physiotherapy for this patient group is time-consuming and requires high staffing levels. Unfortunately, in many health systems, a lack of staff and financial resources prevent early and intensive mobilisation as recommended in many guidelines. Although the intervention investigated is currently not sufficient to replace conventional physiotherapy, it could help to increase the amount of physiotherapy per patient at a very low cost.

VR-based interventions are gaining ground in many different areas of medicine because they are easy to use and often inexpensive, especially compared to interventions that require more medical staff and equipment. Activity-promoting VR applications are not only a potential strategy to reduce sedentary lifestyles but have also been successfully used in neurorehabilitation of stroke patients, patients with paralysis and with Parkinson’s disease to improve cognitive functions, motor skills and balance [40]. Activity-promoting video games can be played on various systems such as gaming consoles, PCs and other devices. VR-based interventions with head-mounted devices can be performed in any location without the need to move the equipment or move the patient to another room, potentially providing a greater degree of flexibility. Currently, there is no data on the postoperative use of VR-based activity-enhancing video games to facilitate physical rehabilitation. Therefore, we initiated a pilot study to investigate the usefulness and feasibility of a VR-based intervention to improve recovery after colorectal cancer surgery.

Since the use of VR-based fitness games in the early postoperative phase could have positive effects on psychological well-being, we decided to assess distress and patient satisfaction in addition to health status as a clinical outcome.

In terms of feasibility, we aim to achieve a minimum participation rate of 70% and a minimum compliance rate of 80% in terms of the completion of questionnaires and participation in the VR sessions offered. Trial participation rates and participation in VR sessions will be analysed during the study. If necessary, the reasons for low attendance rates are analysed and strategies to improve recruitment and participation in the VR sessions offered are discussed with team members.

Although this is a pilot study, we opted for a two-arm design as this provides data from a control, allowing a better understanding of the effect of the study intervention and a more accurate estimate of effect size. Even though randomisation is not a mandatory element of pilot trials we decided to use randomisation to further improve data quality and reduce selection bias. Even though the exercises could be performed by the patient without assistance, we chose to use supervision, to ensure that all exercises are performed correctly, to guarantee adherence to the protocol and to make sure that patients have no problems with the handling of the VR headset. Depending on the results, supervision could be omitted in future trials, if patients are adequately introduced to the system. This could provide an even greater degree of flexibility. The trial intervention could be performed after any abdominal surgery, but we decided to focus on colorectal cancer patients as colorectal cancer surgery is a common procedure and we wanted to ensure a relatively homogeneous patient population.

The proposed pilot study will be the first step to evaluate the feasibility of VR-based interventions in the perioperative period, with the aim of improving the postoperative rehabilitation of cancer patients.

### Trial status

The trial has been registered in the German Clinical Trials Register (DRKS) Nr. DRKS00024888, on April 13, 2021. The full WHO trial registration dataset is available through the WHO ICTRP search portal. The protocol version is 2020.08, Recruitment began on May 12, 2021. The expected date for recruitment completion is March 2022.

## Supplementary Information

**Additional file 1: Supplementary file 1.**

## Data Availability

Not applicable.

## Declarations

### Dissemination

Study findings will be submitted for publication in peer-reviewed journals.
